# Can short-term effectiveness of anti-pronation taping predict the long-term outcomes of customized foot orthoses: developing predictors to identify characteristics of patients with plantar heel pain likely to benefit from customized foot orthoses

**DOI:** 10.1186/s12891-019-2648-3

**Published:** 2019-05-31

**Authors:** Fu-Lien Wu, Yi-Fen Shih, Si-Huei Lee, Hong-Ji Luo, Wendy Tzyy-Jiuan Wang

**Affiliations:** 1 Department of Physical Therapy and Assistive Technology, National Yang-Ming University, No.155, Sec.2, Linong Street, Taipei, 112 Taiwan, Republic of China; 2 Department of Physical Medicine and Rehabilitation, Taipei Veterans General Hospital, Taipei, Taiwan

**Keywords:** Clinical decision, Prediction, Prognostic factors, Foot orthoses, Taping

## Abstract

**Background:**

Foot orthoses are widely used to manage plantar heel pain (PHP). However, the evidence concerning the effect of foot orthoses on PHP is not conclusive. The study aims to identify the characteristics of patients with PHP likely to achieve a positive outcome after customized foot orthoses and to verify the concept that patients who respond positively to anti-pronation taping would achieve a positive prognosis after wearing foot orthoses for six months.

**Methods:**

This is a prospective observational cohort study. Seventy-four patients with PHP underwent a baseline examination and received anti-pronation taping to their painful feet. The taping effects on pain and function were assessed at the 7-day follow-up visit. Then, all patients received an intervention for their PHP with customized foot orthoses for six months. Outcome was assessed with a numeric pain rating scale, the patient-specific functional scale, the foot function index, and the global rating of perceived change. Significant reduction of pain, increase of function, and perception of a meaningful improvement were considered a positive response.

**Results:**

Of 74 patients, 49 had a positive response to the customized foot orthosis treatment. Five predictors were identified: (1) the average pain intensity decreased by over 1.5 points with taping, (2) the range of ankle plantarflexion > 54 degrees, (3) the strength of ankle plantarflexors on the symptomatic side was equal to or stronger than that on the other side, (4) the range of hip internal rotation < 39 degrees, and (5) the range of hip external rotation > 45 degrees. The presence of three or more predictors increased the rate of achieving positive outcome from 66 to 89%.

**Conclusions:**

The predictors of customized foot orthosis outcome in patients with PHP are related to several physical measures of a lower extremity. Findings of the study can be used to screen and select patients with PHP for foot orthosis intervention. Moreover, patients who respond positively to anti-pronation taping would also benefit from the customized foot orthoses. However, since there was no control group in the current study, it is inappropriate to draw conclusions about the effectiveness of the foot orthoses treatment.

**Trial registration:**

The trial was retrospectively registered with the Australian and New Zealand Clinical Trials Registry (ACTRN 12617000119392).

## Background

Plantar heel pain (PHP) is a highly prevalent foot disorder, accounting for 8–20% of both nonathletic and athletic populations [1–3]. First-step pain occurring after prolonged sitting or lying is the most disturbing symptom in patients with PHP [4]. Poor shock absorption of the plantar fascia in supporting body weight is a common contributing factor in explaining the pathomechanism of the PHP [5].

Multiple extrinsic and intrinsic predisposing factors regarding PHP have been reported, inclusive of excessive foot pronation, prolonged weight-bearing activity, limited range of ankle dorsiflexion, unfavorable environment for shock absorption, high body mass index and history of previous lower limb trauma [4, 6–9]. Excessive pronation in foot posture has been viewed as an important biomechanical finding in patients with PHP [4, 6]. Foot orthoses are commonly used to provide a biomechanical anti-pronation support to realign a more pronated foot posture. The strain over the plantar fascia might be attenuated by reducing excessive uncontrolled foot pronation with foot orthoses, resulting in decreasing symptoms of PHP [10, 11]. Despite both customized and prefabricated foot orthoses providing positive short-term (< 3 months) effects in decreasing foot pain and improving function [4, 11], the long-term (> 3 months) effects have not been well studied [12, 13]. One possible reason for the limited evidence on long-term effects of foot orthoses may be related to the heterogeneous subject populations in different studies. Not every patient with PHP benefits from foot orthoses. Hence, identifying subgroups of patients who respond positively to foot orthoses in order to improve the treatment efficacy is worth investigating.

Vicenzino et al. proposed the concept of a ‘treatment direction test (TDT)’ to identify patients who respond to using foot orthoses in treatment. They claimed that anti-pronation taping could be used to assess and determine the effectiveness of wearing foot orthoses [14–16]. One case report and one case series study verified this concept [14, 16]. However, because of the small sample sizes of these two studies, solid evidence for supporting the theory that patients with PHP who are responders to anti-pronation taping would also benefit from foot orthosis intervention has not been confirmed. In order to achieve best practice intervention, it would be prudent to determine predictors associated with successful response to treatment of foot orthoses.

In the past two decades, several studies have identified clinical predictors to enhance clinical professionals’ decision-making process in managing many musculoskeletal disorders, such as low back pain, patellofemoral pain syndrome and neck pain, by classifying patients into specific treatment-based subgroups [17–19]. However, the evidence with regard to predictive factors related to customized foot orthoses for treating PHP was insufficient. Therefore, this study aimed to identify the characteristics of patients with PHP likely to benefit from customized foot orthoses and to verify the concept that patients who respond positively to anti-pronation taping would also benefit from the customized foot orthoses.

## Methods

### Subjects

This is a prospective observational cohort study of 74 patients diagnosed with plantar heel pain by a physiatrist in a general hospital. Inclusion criteria for participants consisted of age older than 20 years, a symptom over the plantar heel or the plantar fascia area, a pain score of 3 points or high on an 11-point (0–10) numeric rating scale (NRS), and a symptom duration of at least 4 weeks. Patients were excluded when they had one of the following situations: (1) symptoms associated with the neurological system, (2) having lower extremity trauma in the past six months, (3) receiving lower extremity surgery in the past six months, or (4) had received corticosteroid injections or other interventions (including taping and foot orthoses) for PHP in the past three months. Before participating in this study, all patients signed an informed consent form that was approved by the Institutional Review Board of Taipei Veterans General Hospital (2013–01-026B). This trial was retrospectively registered with the Australian and New Zealand Clinical Trials Registry (ACTRN12617000119392).

### Examination

To record stable baseline symptoms, we advised all patients to maintain but not to increase their usual activity level at least 2 weeks before the baseline examination. Both a standardized history taking and physical examination were performed on all patients by an experienced physical therapist. A numeric rating scale (NRS) was used to assess the severity of pain [20]. The Foot Function Index (FFI) [21] and Patient-specific Functional Scale (PSFS) [22] were used for quantification of activity limitation and functions. The FFI is a self-reported 23-item questionnaire containing three domains: disability (nine items), activity limitation (five items), and pain (nine items) for evaluating multiple foot and ankle problems [21]. The range of FFI score is between 0 and 100, with a higher score representing more severe pain, disability or functional restrictions [23]. PSFS was assessed with the method of instructing patients to describe three activities they were incapable of performing, or which were difficult to execute because of symptoms. Each patient then self-scored the easiness of each activity on a scale from 0 to 10. Lower scores indicate more difficulty in performing the activity [22].

The passive range of motion of each lower limb joint was measured with a goniometer [24–26]. The isometric muscle strength of the lower limb was assessed with a MicroFET 2 dynamometer (Hoggan Health Industries, Inc., Draper, UT) [27, 28]. Other measurements including bony alignment and special tests [24, 29–35] are presented in Table 2 and Table 3. We recorded all physical examination variables on the symptomatic side (or the most affected side if both sides were involved) and the contralateral side. The between-side difference of each variable was then calculated by subtracting the value of the contralateral side from the value of the symptomatic side. The Foot Posture Index (FPI) containing six validated and observable items was used to quantify the relaxed standing foot posture [36].

### Intervention

**Fig. 1:**
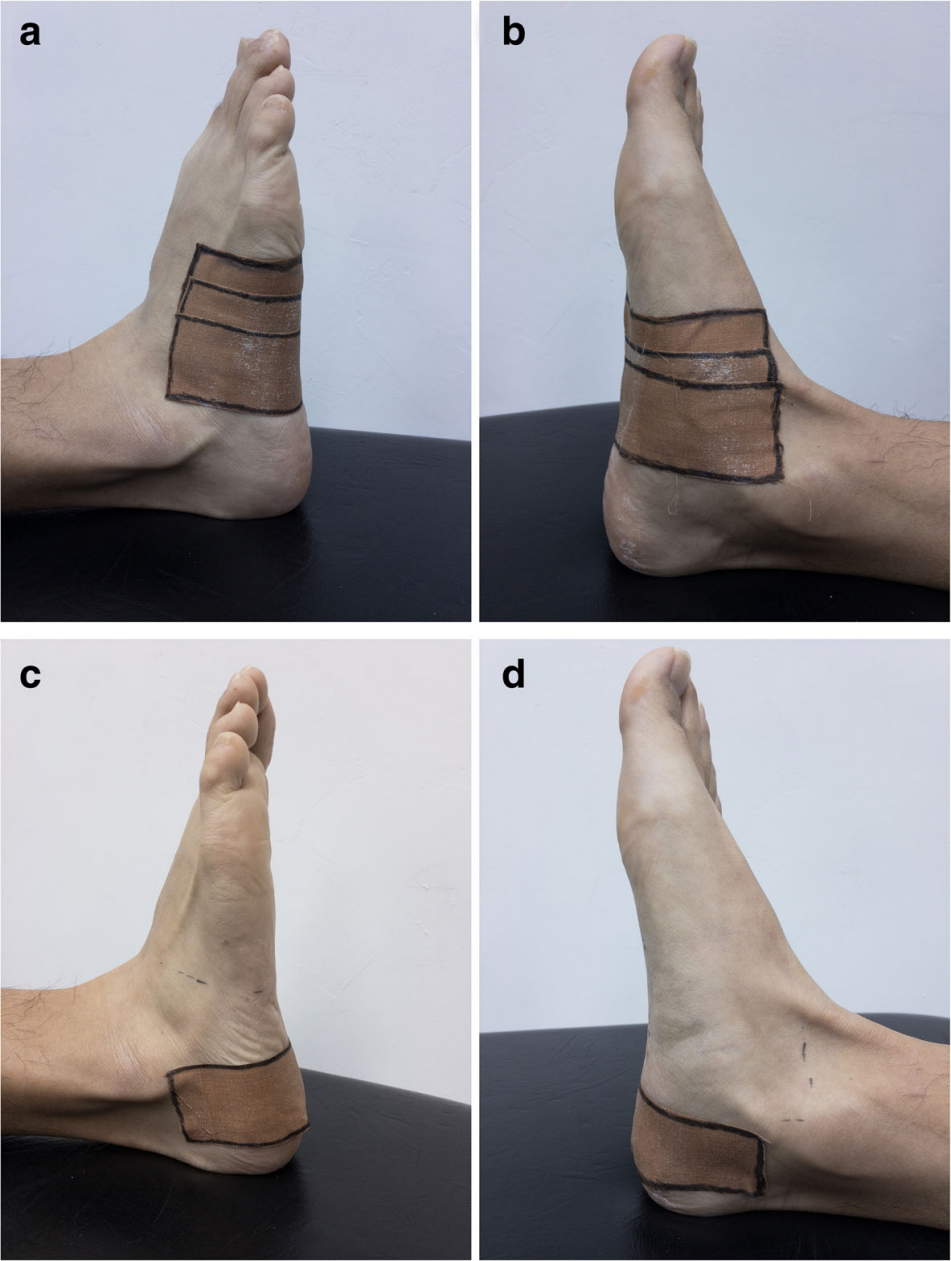
Biomechanical anti-pronation taping includes 2 elements, arch taping and calcaneal taping. (**a**) Arch taping, lateral view. (**b**) Arch taping, medial view. (**c**) Calcaneal taping, lateral view. (**d**) Calcaneal taping, medial view

In order to determine the predictive value of the taping effect on the response from foot orthoses, all patients received customized biomechanical anti-pronation taping (BAPT) before wearing the foot orthoses. BAPT was applied with rigid adhesive tapes (3.8 cm Leuko Sports Tape, Beiersdorf Australasia Ltd., North Ryde, Australia) by a physical therapist. The BAPT method consisting of two taping components, the arch taping and the calcaneal taping, is depicted in Fig. 1 [32, 37, 38]. Each patient was instructed to keep the tape on for 2 to 3 days. All patients were advised to continue their usual activities without exacerbating their symptoms and to avoid pain medications prior to the follow-up visit which was arranged 1 week later.

At the follow-up visit for taping effect, patients reported the changes over the period of BAPT treatment by completing the NRS for pain, FFI, PSFS and the global rating of change (GROC) scale. The GROC scale is a self-administered measure which can be utilized to evaluate the individual’s perception of change after a treatment [39, 40]. Range of the GROC is from + 7 (greatest improvement) to zero (no change) to − 7 (greatest deterioration). The score of GROC ≥ + 4 has been viewed as the cutoff point of a considerable improvement following an intervention [39].

After being assessed for the effectiveness of BAPT, all patients received an individualized foot orthosis intervention for six months. A pair of heat-moldable customized foot orthoses (Vasyli Custom Orthotics; Vasyli International, Labrador, Australia) was given to each patient. All patients were instructed to maintain their usual activities within the limits of symptoms and to wear the foot orthoses for as much time as possible in their day-to-day activities. Subjects were asked to return for follow-up six months later. The response of the foot orthoses was evaluated with the NRS for pain, PSFS, FFI for function, and the GROC scale for perceived effect at the 6-month follow-up visit. The assessor and treatment provider was the same physical therapist.

### Determination of treatment success

At the 6-month follow-up visit, patients would be classified into either the success or nonsuccess group based on their reported outcomes. The criteria for inclusion of the success group were: (1) reducing the pain intensity by more than two points [20] or more than 50%, (2) decreasing the FFI score by more than seven points or improving the FFI or PSFS score by more than 50% [23], and (3) reporting an overall change on the GROC scale of + 4 or higher [39]. All patients in the success group had to meet all three criteria.

### Data analysis

According to the rule of thumb recommended by Peduzzi et al. [41] and Harrell et al. [42], 10 outcome events were required for each predictor variable that was entered into a multiple logistic regression. The sample size for this study was estimated to include seven to eight potential predictors in the final regression model, therefore, we aimed to recruit 70 to 80 patients with PHP.

We used SPSS version 20.0 (IBM Corp., Armonk, NY, USA) to determine whether any potential predictor variables and anti-pronation taping response identified patients who benefited from the 6-month foot orthosis treatment. Firstly, univariate analyses including independent t tests for continuous variables and chi-square tests for categorical variables were used to determine the significant differences of independent variables between the success and the nonsuccess groups. The method to derive the optimal cutoff point of each significant continuous variable in the independent t test was the Receiver Operating Characteristic (ROC) curve. Then the variables that demonstrated significant between-group differences at *p* < 0.15 were entered into a multiple logistic regression to derive significant predictors for a positive outcome from the use of customized foot orthoses. A significance level of 0.05 was used for this multiple logistic regression.

## Results

**Fig. 2:**
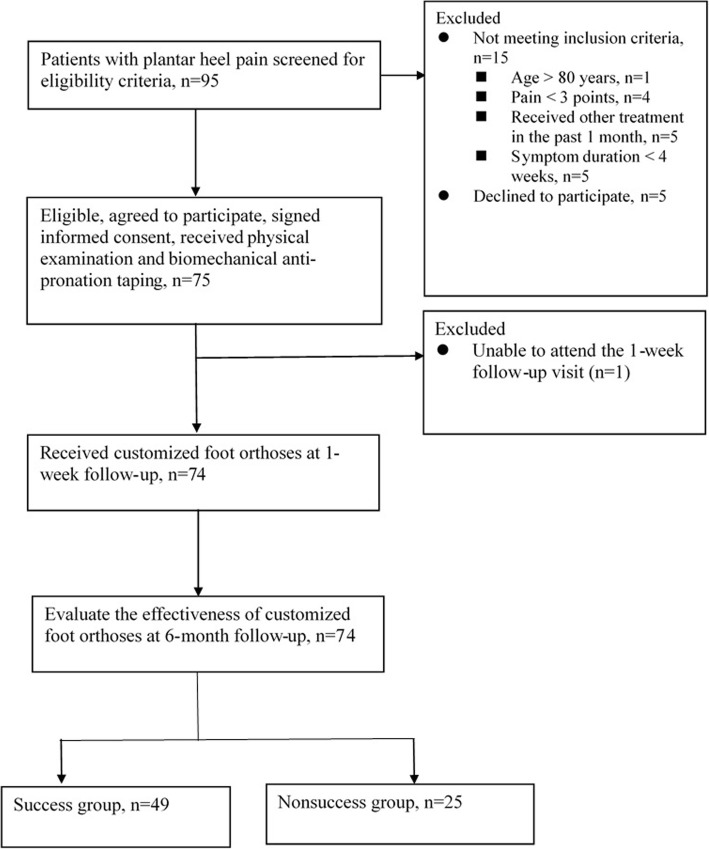
Flow diagram of the study

Seventy-five patients with PHP participated in this study from March 2013 to June 2016. One patient dropped out from the study due to the incapability of returning for the first-week follow-up. The average age of all subjects was 48.4 ± 14.5 years. The process of recruiting and retaining subjects is shown in Fig. 2. According to the criteria of judging the positive outcome, 49 patients (66%) were classified into the success group and 25 patients (34%) into the nonsuccess group. Table 1 provides the baseline data of patients with successful and unsuccessful outcomes. The self-reported measures and the physical examination variables for the study participants are presented in Tables 2, 3, 4 to 5.

**Table 1:** Baseline demographic data

Variable	All Subjects (*n* = 74)	Success (*n* = 49)	Nonsuccess (*n* = 25)	*p* value
Age, year	48.4 ± 14.5	49.0 ± 14.1	47.2 ± 14.6	0.630
Women, n (%)	57 (77.0%)	38 (77.6%)	19 (76.0%)	1.000
Body mass index, kg/m^2^	23.8 ± 3.7	24.1 ± 3.6	23.4 ± 4.0	0.453
Duration of symptom, month	30.3 ± 55.7	29.3 ± 45.1	32.1 ± 73.4	0.839
First-step pain NRS	4.6 ± 2.7	4.7 ± 2.7	4.3 ± 2.8	0.599
Usual pain NRS	4.9 ± 1.8	5.1 ± 1.7	4.4 ± 1.9	0.122*
PSFS	4.6 ± 1.5	4.6 ± 1.5	4.5 ± 1.6	0.810
FFI	34.3 ± 17.6	35.2 ± 16.2	32.5 ± 20.3	0.533
Weekly working time, hour	32.7 ± 22.4	32.7 ± 23.0	32.8 ± 21.6	0.992
Daily weight-bearing time, hour	3.1 ± 2.9	3.0 ± 2.6	3.3 ± 3.3	0.672
Dominant leg: right, *n* (%)	67 (90.5%)	43 (87.8%)	24 (96.0%)	0.411
Mode of onset: gradual, *n* (%)	48 (64.9%)	30 (61.2%)	18 (72.0%)	0.567
Episode: first, *n* (%)	44 (59.5%)	29 (59.2%)	15 (60.0%)	1.000

*p < 0.15

*NRS* numeric rating scale, *PSFS* patient-specific functional scale, *FFI* foot function index

Data present as mean ± SD for continuous variables and percent for categorical variables

**Table 2:** Baseline measurement of the foot and ankle joint

Variable	All Subjects (*n* = 74)	Success (*n* = 49)	Nonsuccess (*n* = 25)	*p* value
Rearfoot varus in STNP, deg	7.3 ± 2.5	7.0 ± 2.3	7.8 ± 2.7	0.227
Forefoot varus in STNP, deg	10.2 ± 4.2	10.7 ± 3.2	9.2 ± 5.7	0.141*
Rearfoot inversion ROM, deg	21.8 ± 6.1	21.5 ± 7.3	20.9 ± 5.6	0.649
Rearfoot eversion ROM, deg	9.3 ± 4.8	9.1 ± 5.1	9.5 ± 4.2	0.733
1st MTP joint extension ROM, deg	69.3 ± 11.5	68.7 ± 10.2	70.6 ± 13.9	0.505
Ankle dorsiflexion ROM, deg	10.8 ± 7.1	10.5 ± 7.1	11.6 ± 7.1	0.536
Ankle plantarflexion ROM, deg	53.1 ± 12.1	54.7 ± 11.0	50.1 ± 13.7	0.122*
Navicular drop test,^34^ mm	10.9 ± 4.6	11.0 ± 4.6	10.8 ± 4.7	0.893
Calcaneus valgus in RCSP,^35^ deg	6.0 ± 5.0	6.1 ± 5.0	5.9 ± 5.2	0.867
Calcaneus valgus in NCSP,^35^ deg	−4.7 ± 3.5	−4.9 ± 3.2	− 4.2 ± 4.1	0.437
Foot posture index	6.3 ± 3.7	6.2 ± 3.8	6.5 ± 3.5	0.715

*p < 0.15

*STNP* Subtalar joint neutral position, *ROM* Range of motion, *MTP* Metatarsophalangeal, *RCSP* Relaxed calcaneal stance position, *NCSP* Neutral calcaneal stance position

Data present as mean ± SD

**Table 3:** Baseline measurement of the hip and knee joints

Variable	All Subjects (*n* = 74)	Success (*n* = 49)	Nonsuccess (*n* = 25)	*p* value
Femoral neck anteversion, ^27^ deg.	7.7 ± 5.8	7.5 ± 6.3	8.0 ± 4.5	0.750
Tibial lateral torsion,^32^ deg	17.9 ± 7.0	18.1 ± 7.2	17.7 ± 6.9	0.825
Leg length difference,^35^ mm	1.0 ± 6.5	1.0 ± 5.8	1.1 ± 7.8	0.716
Quadriceps angle,^34^ deg	14.3 ± 5.7	14.7 ± 4.9	13.6 ± 7.0	0.452
Hip internal rotation ROM, deg	35.8 ± 13.1	33.5 ± 12.4	40.4 ± 13.4	0.030*
Hip external rotation ROM, deg	47.2 ± 12.8	49.2 ± 12.7	43.3 ± 12.3	0.060*
Straight leg raise range,^36^ deg	58.9 ± 14.1	57.2 ± 13.8	62.2 ± 14.4	0.157
Hip flexion ROM in Thomas test,^37^ deg	−19.2 ± 9.5	−19.5 ± 9.4	− 18.6 ± 9.8	0.718
Knee flexion ROM in Thomas test,^37^ deg	50.0 ± 14.5	50.0 ± 14.0	50.0 ± 15.7	0.999
Hip adduction ROM in Ober’s test,^38^ deg	14.5 ± 9.2	13.6 ± 10.1	16.4 ± 6.7	0.221

*p < 0.15

*ROM* Range of motion

Data present as mean ± SD

**Table 4:** Baseline isometric muscle strength normalized with body weight (%)

Variable	All Subjects (n = 74)	Success (*n* = 49)	Nonsuccess (*n* = 25)	*p* value
Ankle dorsiflexor	26.4 ± 7.4	25.8 ± 6.7	27.5 ± 8.7	0.356
Ankle dorsiflexor (between-side difference)	0.1 ± 3.7	0.1 ± 3.6	0.1 ± 3.8	0.956
Ankle plantarflexor	34.9 ± 13.0	35.0 ± 13.0	35.0 ± 13.3	0.814
Ankle plantarflexor (between-side difference)	−0.3 ± 5.3	0.8 ± 5.2	−2.4 ± 4.8	0.011*
Ankle invertor	14.9 ± 5.9	14.5 ± 5.7	15.7 ± 6.4	0.420
Ankle invertor (between-side difference)	−0.5 ± 3.4	− 0.6 ± 3.4	− 0.4 ± 3.6	0.857
Ankle evertor	15.1 ± 6.3	14.7 ± 6.1	15.9 ± 6.8	0.444
Ankle evertor (between-side difference)	−0.7 ± 11.2	0.7 ± 2.9	−3.4 ± 18.8	0.296
1st toe flexor	8.7 ± 2.9	−0.5 ± 9.4	4.8 ± 9.2	0.896
1st toe flexor (between-side difference)	−0.2 ± 1.6	8.7 ± 3.0	8.8 ± 2.8	0.714
Knee flexor	15.9 ± 4.8	16.3 ± 4.4	15.2 ± 4.9	0.357
Knee flexor (between-side difference)	−0.3 ± 1.5	− 0.3 ± 1.6	−0.2 ± 1.2	0.634
Knee extensor	25.5 ± 10.1	26.5 ± 11.3	23.7 ± 7.0	0.200
Knee extensor (between-side difference)	2.2 ± 7.3	2.1 ± 8.9	2.3 ± 2.0	0.874
Hip flexor	35.3 ± 7.1	22.0 ± 5.9	21.2 ± 5.9	0.585
Hip flexor (between-side difference)	0.4 ± 2.9	0.7 ± 3.1	−0.0 ± 2.4	0.348
Hip extensor	25.9 ± 8.3	25.6 ± 8.1	26.5 ± 8.7	0.643
Hip extensor (between-side difference)	0.6 ± 2.4	0.5 ± 2.6	0.8 ± 1.9	0.598
Hip abductor	19.3 ± 4.1	18.9 ± 3.9	20.0 ± 4.4	0.289
Hip abductor (between-side difference)	−0.1 ± 2.7	−0.3 ± 2.7	0.3 ± 2.8	0.385

*p < 0.15

Data present as mean ± SD

**Table 5:** Outcome measures after biomechanical anti-pronation taping

Variable	All Subjects (*n* = 74)	Success (*n* = 49)	Nonsuccess (*n* = 25)	*p* value
First-step pain NRS	3.0 ± 2.5	3.0 ± 2.7	3.1 ± 2.3	0.924
Change in first-step pain NRS	−1.5 ± 2.2	−1.7 ± 2.4	−1.2 ± 1.7	0.395
Usual pain NRS	3.1 ± 2.0	2.9 ± 2.1	3.3 ± 1.6	0.438
Change in usual pain NRS	−1.8 ± 1.9	−2.1 ± 2.0	− 1.1 ± 1.6	0.023*
PSFS	6.3 ± 1.8	6.4 ± 1.9	6.3 ± 1.6	0.812
Change in PSFS	1.8 ± 1.6	1.8 ± 1.5	1.8 ± 1.8	0.975
FFI	24.6 ± 19.8	23.3 ± 19.8	27.0 ± 20.1	0.462
Change in FFI	−9.7 ± 14.8	−11.9 ± 15.6	−5.6 ± 12.3	0.082*
GROC	3.2 ± 1.9	3.4 ± .0	3.00 ± 1.8	0.456

*p < 0.15

*NRS* Numeric rating scale, *PSFS* Patient-specific functional scale, *FFI* Foot function index, *GROC* Global rating of changes

Acquisition of change score of each variable is by subtracting the baseline value from the posttest value of each outcome measure after taping

Data present as mean ± SD

Eight potential demographic, self-reported and physical examination variables which demonstrated significant between-group differences in the univariate analyses were retained as prediction variables. The accuracy statistics and 95% confidence intervals (CIs) for all eight potential predictors entering into the logistic regression are presented in Table 6. The final multiple logistic regression identified five predictors (Table 7) including the average pain intensity decreased by over 1.5 points after taping, the range of ankle plantarflexion > 54 degrees, the strength of ankle plantarflexors on the symptomatic side was equal to or stronger than that on the contralateral side, the range of hip internal rotation < 39 degrees, and the range of hip external rotation > 45 degrees (Hosmer-Lemeshow chi-square = 8.9, df = 8, *p* = 0.347, Nagelkerke R square = 0.511).

**Table 6:** Accuracy statistics (95% confidence intervals) for potential predictors (pre-test rate of success 66%)

Variable	Sensitivity	Specificity	Positive likelihood ratio	Posttest probability
Baseline pain > 4.5 points	0.59 (0.44, 0.73)	0.48 (0.28, 0.69)	1.14 (0.73, 1.77)	69% (59, 75%)
Forefoot varus > 10 degrees	0.69 (0.55, 0.82)	0.36 (0.18, 0.57)	1.08 (0.77, 1.54)	68% (60, 75%)
Ankle plantarflexion ROM > 54 degrees	0.55 (0.40, 0.69)	0.60 (0.39, 0.79)	1.38 (0.80, 2.37)	73% (61, 82%)
Hip internal rotation ROM < 39 degrees	0.57 (0.42, 0.71)	0.68 (0.47, 0.85)	1.79 (0.96, 3.32)	78% (65, 87%)
Hip external rotation ROM > 45 degrees	0.73 (0.59, 0.85)	0.52 (0.31, 0.72)	1.53 (0.98, 2.38)	75% (66, 82%)
Ankle plantarflexors strength (the symptomatic side ≥ the contralateral side)	0.69 (0.55, 0.82)	0.68 (0.47, 0.85)	2.17 (1.19, 3.95)	81% (70, 89%)
Average pain intensity decreased by over 1.5 points with taping	0.69 (0.55, 0.82)	0.68 (0.47, 0.85)	2.17 (1.19, 3.95)	81% (70, 89%)
FFI score decreased by over 8 points with taping	0.65 (0.50, 0.78)	0.64 (0.43, 0.82)	1.81 (1.04, 3.18)	78% (67, 86%)

*ROM* Range of motion, *FFI* Foot function index

**Table 7:** Prevalence of individual and grouped prognostic factors at each level^*^

Variable	Success group (*n* = 49)	Nonsuccess group (*n* = 25)
5	3	0
4+	17	1
3+	42	5
2+	48	17
1+	49	23

*Predictors: the average pain intensity decreased by over 1.5 points with taping, ankle plantarflexion ROM > 54 degrees, the strength of ankle plantarflexors on the symptomatic side equal to or stronger than that on the contralateral side, hip internal rotation ROM < 39 degrees, and hip external rotation ROM > 45 degrees

The pre-test rate of success with 6-month foot orthosis intervention was 66%. Considering the current clinical predictors, if the patient exhibited at least three predictors, the positive likelihood ratio (+LR) was 4.3 (95% CI: 1.9, 9.5) with an 89% (95% CI: 79, 99%) post-test rate of success (Table 8). If a patient exhibited fewer than three criteria of this prediction model, the post-test rate of success would be below 80%.

**Table 8:** Accuracy statistics for 5 levels of the clinical predictors* (95% confidence intervals)

Variable	Sensitivity	Specificity	Positive likelihood ratio	Posttest probability
5	0.06 (0.01, 0.17)	1.00 (0.86, 1.00)	inf (inf, inf)	100% (100, 100%)
4+	0.35 (0.22, 0.50)	0.96 (0.80, 1.00)	8.67 (1.22, 61.48)	94% (71, 99%)
3+	0.86 (0.73, 0.94)	0.80 (0.59, 0.93)	4.29 (1.94, 9.46)	89% (79, 95%)
2+	0.98 (0.89, 1.00)	0.32 (0.15, 0.54)	1.44 (1.10, 1.89)	74% (68, 79%)
1+	1.00 (0.93, 1.00)	0.08 (0.01, 0.26)	1.09 (0.97, 1.22)	68% (65, 71%)

inf: infinity

*Predictors: the average pain intensity decreased by over 1.5 points with taping, ankle plantarflexion ROM > 54 degrees, the strength of ankle plantarflexors on the symptomatic side equal to or stronger than that on the contralateral side, hip internal rotation ROM < 39 degrees, and hip external rotation ROM > 45 degrees

## Discussion

This study develops a clinical prediction rule that identified several clinically measurable characteristics to predict a positive prognosis after a 6-month foot orthosis intervention for patients with PHP. The rate of success could increase from 66 to 89% with a + LR of 4.3 if a patient exhibited three of the following five predictors: (1) the average pain intensity decreased by over 1.5 points after BAPT, (2) the range of ankle plantarflexion > 54 degrees, (3) the strength of ankle plantarflexors on the symptomatic side was equal to or stronger than that on the contralateral side, (4) the range of hip internal rotation < 39 degrees, and (5) the range of hip external rotation > 45 degrees.

The first predictive factor emerged from the final logistic regression model was that the average pain intensity decreased by over 1.5 points with taping. Based on this result, we verified that patients who respond positively to anti-pronation taping would also benefit from the customized foot orthoses. The decrease in pain with taping can be partly attributed to the reduction of excessive foot pronation during walking. Excessive or prolonged foot pronation during the stance phase of gait has been recognized as a risk factor of developing PHP [4, 6, 9]. One meta-analysis revealed that both anti-pronation taping and foot orthoses significantly decreased the calcaneus eversion angle during standing or walking, thereby causing reduction of foot pronation [10]. Because anti-pronation taping and foot orthoses provide a biomechanical anti-pronation support on the foot to decrease the excessive stress over the plantar fascia, patients with PHP who received either the taping or orthosis intervention should show similar positive responses. Our study results supported the concept of ‘treatment direction test (TDT)’ introduced by Vicenzino et al. [14–16]. The TDT involves using anti-pronation taping to determine if controlling excessive foot pronation would result in a reduction of patients’ pain and discomfort during their physical activity. If a TDT was positive, i.e. the patient showed improvement in pain after receiving anti-pronation taping, we would assume that the patient would benefit from the foot orthoses. From the results of our study, a reduction of pain intensity by over 1.5 points after anti-pronation taping can become an objective indicator to subsequently prescribe foot orthoses.

The second significant predictor for positive outcome was ‘the range of ankle plantarflexion > 54 degrees’. According to a previous study reporting the normative values of joint range of motion, the mean value of ankle plantarflexion was 56.5 degrees for general population [43]. Greater than normal ankle plantarflexion range implies excessive length of the ankle dorsiflexor muscles [44]. Excessive length of a muscle has been associated with over-stretch weakness, which indicates that elongated muscles appear to be less efficient in generating force [45, 46]. The efficiency of an elongated tibialis anterior to eccentrically control foot pronation during walking and running could be affected [44]. Theoretically, elongation of tibialis anterior, associated with greater plantarflexion range, could increase the amount of navicular drop when weight bearing on the foot. Hence, greater plantarflexion range could contribute to excessive foot pronation [44]. The findings of our study provided a clinical implication that patients with PHP who had greater than normal range of ankle plantarflexion were more likely to benefit from the six-month foot orthosis intervention.

The strength of ankle plantarflexors on the symptomatic side equal to or stronger than that on the contralateral side was the third predictor of positive response found in the present study. The ankle plantarflexor strength has been considered as an essential factor contributing to the step length and walking velocity [5, 6]. There were three potential roles of ankle plantarflexor in gait proposed in the previous study [6]: (1) to assist the trunk balance when weight-bearing on the foot, (2) to transfer the lower limb from the stance phase to the swing phase, and (3) to assist the human body moving forward. People who had weak ankle plantarflexor exhibited slower walking speed and shorter step length [5, 6], which significantly associated with lower extremity function. The current result indicated that patients with PHP who had the same or stronger ankle plantarflexors on the symptomatic side still retained appropriate foot function and, therefore had a higher chance to respond to the foot orthosis treatment. This study result also implies that patients with PHP whose ankle plantarflexors strength was less than that of the other side should be recommended to perform strengthening exercises. Rathleff et al. conducted a clinical trial and found that a 3-mouth single heel raising training program significantly decreased pain and improved foot function in patients with PHP [47].

The last 2 significant predictors identified from the current study were both pertaining to the hip mobility. One predictor was ‘the range of hip internal rotation < 39 degrees’, and the other was ‘the range of hip external rotation > 45 degrees’. This clinical presentation with increased external rotation and decreased internal rotation ranges of motion at the hip was found in a study to be a risk factor associated with medial tibial stress syndrome [48]. A possible explanation can be postulated that these changes in hip rotation range of motion caused people to walk with an out-toeing gait which might increase the medial rotation loading on tibia and result in excessive foot pronation. Excessive out-toeing leads to a number of lower extremity problems associated with abnormal foot pronation, such as plantar fasciitis [4, 49] and medial tibial stress syndrome [44, 50–52]. The use of foot orthoses could not only control the foot over-pronation but also exert an influence on the proximal segments of the lower extremity. From the observation in this study, patients with PHP who exhibited these clinical characteristics (increased hip external rotation range and decreased hip internal rotation range) had a higher chance of achieving positive outcome with customized foot orthoses.

There were several limitations in our study. Firstly, because of the use of a single group design, we are unable to infer the effectiveness of foot orthoses for patients with PHP. The second limitation was that the current clinical predictors may not be generalized to the whole population because women accounted for 77% in this study population. More importantly, after identifying the preliminary clinical predictors, these significant predictors must be validated by well-designed randomized controlled trials in future.

## Conclusion

This study identified five significant predictors that determined which patients with PHP would respond positively to customized foot orthoses. These predictors for the effect of customized foot orthosis in patients with PHP are related to several physical measures of a lower extremity. The clinical prediction rule derived from this study can be used to screen and select patients with PHP for foot orthosis intervention. Moreover, we verified the concept that if a patient had a positive ‘treatment direction test’ (i.e. improvement on pain after receiving anti-pronation taping), we could assume this patient would benefit from the foot orthoses intervention. From the current study results, a reduction of pain intensity by over 1.5 points after application of an anti-pronation taping is an objective indicator of favorable outcome following foot orthoses treatment. However, since there was no control group in the current study, it is inappropriate to draw conclusions about the effectiveness of the foot orthoses treatment.

## Data Availability

The datasets used in the present study may be available from the corresponding author on reasonable requests.
